# The limitations of targeting MEK signalling in Glioblastoma therapy

**DOI:** 10.1038/s41598-020-64289-6

**Published:** 2020-05-04

**Authors:** Karthika D. Selvasaravanan, Nicole Wiederspohn, Amina Hadzalic, Hannah Strobel, Christel Payer, Andrea Schuster, Georg Karpel-Massler, Markus D. Siegelin, Marc-Eric Halatsch, Klaus-Michael Debatin, Mike-Andrew Westhoff

**Affiliations:** 1 Department of Pediatrics and Adolescent Medicine, University Medical Center Ulm, Ulm, Germany; 2 Present Address: Interfaculty Institute of Biochemistry, University of Tübingen, Tübingen, Germany; 3 Present Address: Institute for Applied Physiology, University of Ulm, Ulm, Germany; 4 Department of Neurosurgery, University Medical Center Ulm, Ulm, Germany; 5 Department of Pathology and Cell Biology, Columbia University Medical Center, New York, NY USA

**Keywords:** Translational research, Cancer therapy

## Abstract

Glioblastoma (GB) is a highly aggressive, difficult to treat brain tumour. Successful treatment, consisting of maximal safe tumour de-bulking, followed by radiotherapy and treatment with the alkylating agent Temozolomide (TMZ), can extend patient survival to approximately 15 months. Combination treatments based on the inhibition of the PI3K pathway, which is the most frequently activated signalling cascade in GB, have so far only shown limited therapeutic success. Here, we use the clinically approved MEK inhibitor Trametinib to investigate its potential use in managing GB. Trametinib has a strong anti-proliferative effect on established GB cell lines, stem cell-like cells and their differentiated progeny and while it does not enhance anti-proliferative and cell death-inducing properties of the standard treatment, i.e. exposure to radiation or TMZ, neither does MEK inhibition block their effectiveness. However, upon MEK inhibition some cell populations appear to favour cell-substrate interactions in a sprouting assay and become more invasive in the Chorioallantoic Membrane assay, which assesses cell penetration into an organic membrane. While this increased invasion can be modulated by additional inhibition of the PI3K signalling cascade, there is no apparent benefit of blocking MEK compared to targeting PI3K.

## Introduction

Glioblastoma (GB), formerly Glioblastoma multiforme, is the most common primary brain tumour in adults and – due to its aggressive and highly infiltrative nature – among the most lethal malignancies *per se*^1^. The current standard therapy involves maximum safe surgical resection followed by radio- and chemotherapy with the alkylating agent Temozolomide (TMZ)^2^ which leads to a mean patient survival of only 15 months^3^. Although GB only rarely metastasised outside the neuraxis, upon clinical presentation it will almost invariably have infiltrated the surrounding brain tissue, impeding full surgical resection and resulting in tumour recurrence^4^.

GB exhibits a rather low mutational burden^5^, with the most consistent alterations found in the PI3K signalling cascade, which is activated in ~88% of all GBs^6,7^. Despite intensive efforts to translate modulation of this signalling pathway into a clinical setting, so far, only two mTOR (a downstream modulator of PI3K signalling) inhibitors, Everolimus and Temsirolimus, have been approved for clinical use^8^. This might be, at least in part, due to the complexity of the PI3K pathway, while frequently reduced to a “survival pathway” in the context of cancer research, this network has several distinct and somehow competing functions^9,10^. In our hands, inhibition of PI3K signalling in differentiated GB cells (DGBCs) only weakly reduces their proliferation and has little effect on their resistance to apoptosis; however, it strongly reduces their motility. In contrast the motility of the corresponding stem cell-like cells (SCs) was not affected, but the increased resistance of SCs compared to DGBCs with regard to apoptosis induction, seems to be mediated, at least in part, by PI3K signalling^11,12^. Using a dual-kinase inhibitor that blocks PI3K- and mTOR-mediated signalling only slightly increases overall efficacy^13^, therefore, we looked at alternative or additional putative targets that affect GB aggressiveness and could be potentially added to our therapeutic arsenal.

Engelman and co-workers suggested in 2008 that combining PI3K and MEK inhibitors can have a synergistic therapeutic effect in a lung cancer model^14^. Similar data was also presented for melanoma^15^. These findings are not surprising, considering how intrinsically linked the MEK and the PI3K signalling cascades are, exhibiting many putative points of interaction^16–18^.

Similar to the PI3K signalling cascade, the MEK/ERK pathway is considered to enhance survival and confer resistance against radio- and chemotherapy^19,20^, although this seems to be not universally accepted^21,22^. ERK has been implicated in several cellular aspects of GB behaviour, again frequently overlapping with the previously identified roles of the PI3K signalling cascade: The MEK/ERK pathway enhances migration and invasion of GB cells in response to treatment^23^, and increases DNA damage repair both through non-homologous end joining repair as well as homologous recombinational repair pathways^24^. The role of ERK in maintenance of stemness is rather controversial. Depending on the cellular system used, ERK was shown not to contribute to cell proliferation^25^, induce differentiation^26^, or maintain “stemness”^27^. In GB, ERK expression has been associated with SCs^28^, but the interconnectedness of PI3K and ERK signalling often makes it difficult to distinguish between the effects of these signalling cascades on proteins further downstream of the signalling pathways. Therefore, we decided to investigate the role of MEK/ERK signalling in GB using the clinically approved small molecule inhibitor Trametinib.

Trametinib is the first FDA-approved MEK1/2 inhibitor currently in use for unresectable and metastatic melanoma with BRAF V600E or V600K mutation in combination with Dabrafenib^29^. Importantly, Trametinib is a feedback buster inhibitor, that also inhibits the activation of MEK activated Raf, resulting from negative feedback by inactivated MEK. It antagonizes MEK 1 and 2 phosphorylation and promotes the dissociation of RAF-MEK1/2 complexes^30,31^. The clinical advantages of this drug are its long bioavailability (~4 days), rapid absorption after administration (peak plasma concentration within 1.5 hours after administration) and minimal side effects, as well as its effectiveness at very low concentration^32^. Trametinib is currently being clinically evaluated for the treatment of paediatric neuro-oncology patients with refractory tumours (ClinicalTrials.gov Identifier: NCT03363217), including high-grade glioma (ClinicalTrials.gov Identifier: NCT02684058).

## Results

### Trametinib exerts a strong antiproliferative effect on Glioblastoma (GB) cell lines

**Figure 1:**
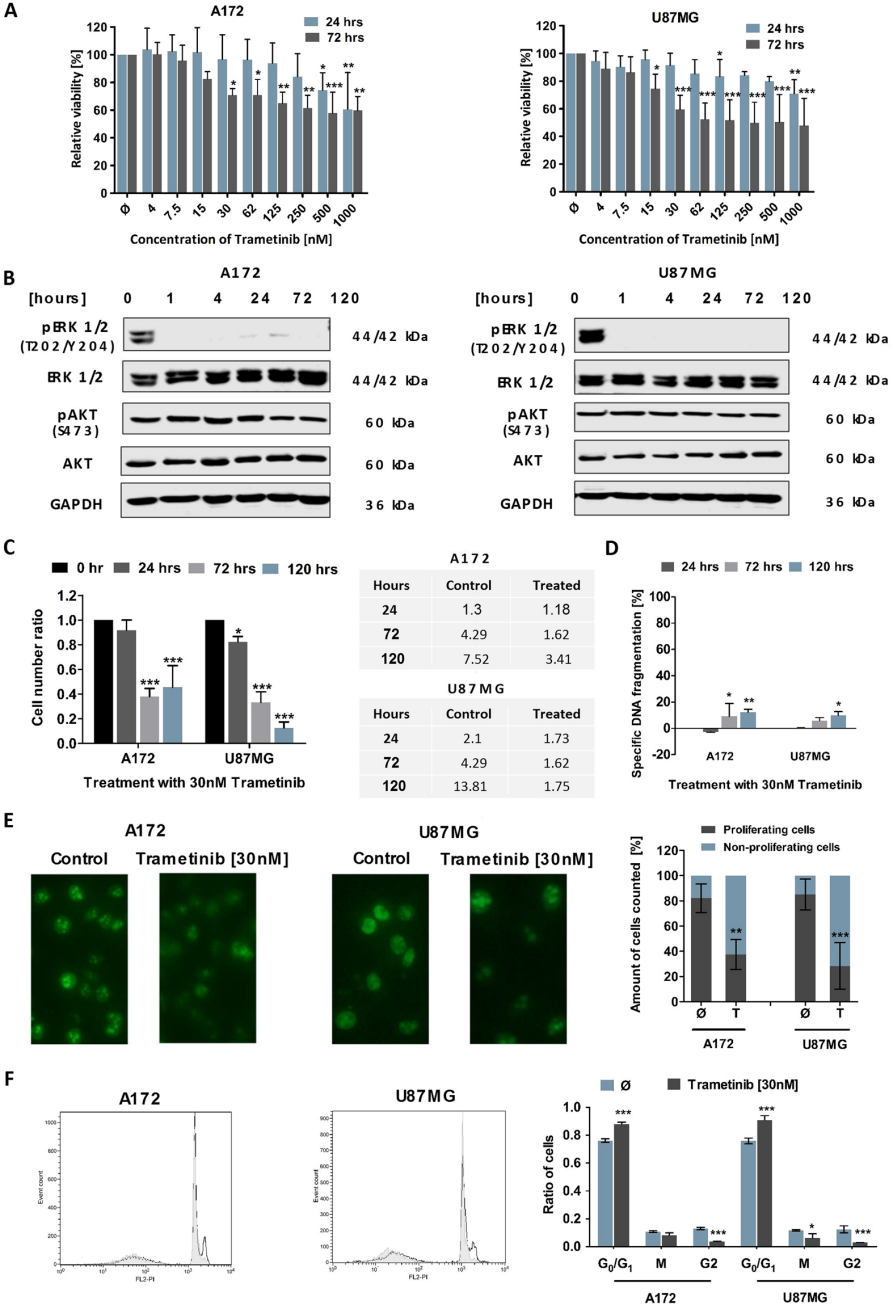
The effect of the MEK inhibitor Trametinib on the two established GB cell lines, A172 and U87MG. (**A**) Effect of Trametinib on cell viability of GB cell lines. Shown are the MTT assay results for the A172 (left) and U87MG (right) cell lines. The cells were treated with indicated concentrations of Trametinib and the metabolic activity was measured after 24 and 72 hours. Data was normalized to the DMSO-treated control. (**B**) Effect of Trametinib on signalling proteins in GB cell lines. Activity of the MEK and PI3K signalling cascades was assessed by Western blot analysis respectively using phosphorylation of ERK and Akt as surrogate readouts. The GB cell lines A172 (left) and U87MG (right) were treated with 30 nM of the MEK inhibitor Trametinib for the indicated times. GAPDH served as loading control. (**C**) Effect of Trametinib on cell number in GB cell lines. The number of viable A172 and U87MG cells was measured using a cell counter at 24, 72 and 120 hours after treatment with 30 nM Trametinib. The control cells were treated with DMSO. The cell number ratio was defined as the ratio of cell number in treated population to cell number in the respective control, i.e. a ratio of 1 indicates no effect, whereas a ratio of 0.5 means treatment resulted in a 50% decrease of cell number compared to control cells at the same time point. The cell numbers at 0 hour were considered to be equal for the control and treated and hence taken as 1. Absolute cell Absolute cell number ×10^4^ are also shown. (**D**) Effect of Trametinib on induction of apoptosis in GB cell lines. Flow cytometric analysis of Propidium-iodide stained nuclei after 24, 72 and 120 hours of treatment with 30 nM of Trametinib in A172 and U87MG cells. Control cells were treated with DMSO. Treatment-induced DNA fragmentation (specific apoptosis) was calculated as described in Materials and Methods. (**E**) Effect of Trametinib on proliferation of GB cell lines. Exemplary pictures of Ki-67-stained A172 and U87MG cells after treatment with 24 hours exposure to Trametinib; C: control cells, T: Trametinib-treated cells (left). Quantitative analysis of Ki-67-stained (proliferating) and Ki67 negative (non-proliferating) cells. (**F**) Effect of Trametinib on cell cycle distribution. Exemplary histograms of the cell cycles of A172 and U87MG cells after 24 hours of exposure to solvent (white) or Trametinib (grey) on the left. Corresponding quantitative analysis of the cell cycle on the right. In (**A,D**) mean and +SD of three independent experiments performed in sextuplicate are shown, in (**B**) one representative result of the three independent experiments is shown, in (**C**) mean and SD of three independent experiments performed in triplicate are shown, in (**E**) mean and +SD of three independent experiments are shown (at least 100 cells were counted for each experimental condition), while in (**F**) three technical repeats of a representative experiment out of three independent replications were analysed and are shown as mean and SD. Statistical significance was assessed by ANOVA (*p-value < 0.05; **p-value < 0.01; ***p-value < 0.001).

First, we evaluated the effect of the highly specific MEK1/2 inhibitor Trametinib on the metabolic activity of two established GB cell lines, A172 and U87MG (Fig. 1A). As Trametinib exposure at a concentration of 30 nM led to a significant reduction of metabolic activity within the first 72 hours, we decided to use this clinically relevant concentration for our further experiments. Western blot analysis confirmed that phosphorylation of ERK1/2, a direct downstream target of MEK and therefore a good surrogate readout for MEK activity, was potently reduced, starting prior to an hour and lasting at least until 120 hours following exposure to the drug (Fig. 1B). Of note, phosphorylation of Akt, a central downstream mediator of PI3K-induced signalling, remained unaffected during the observed time period, suggesting that PI3K signalling does not compensate for reduced MEK signalling in the observed experimental system (Fig. 1B).

To assess whether the reduction in metabolic activity was also reflected in a therapeutically relevant readout, we next looked at cell numbers and found that when assessing the ratio of Trametinib-treated cells to control cells, that ratio declined over time. Comparing, however, absolute cell numbers, treated cells remained at a relatively constant level over time, while the absolute number of control cells increased, which indicates proliferation and was to be expected (Fig. 1C). These findings therefore suggest a cytostatic rather than a cytotoxic effect of Trametinib. This is further supported by the fact that only ~10% DNA fragmentation occurred during the relevant time period (Fig. 1D), i.e. Trametinib on its own only weakly induces apoptosis. The antiproliferative effect on A172 cells appears less pronounced than on U87MG cells, as, at a later time point between 72 and 120 hours after treatment, the A172 cells exposed to Trametinib slowly begin to proliferate again. This happens concurrently with the detection of faint phospho-specific bands in the Western blot analysis of ERK (compare Fig. 1B,C). However, it should be pointed out that no re-exposure to Trametinib occurred, all cells were only treated once with the drug, supporting the previously described durability of this inhibitor^33^. To further confirm our hypothesis that Trametinib works predominantly in a cytostatic manner on GB cells, we next looked at the expression of Ki67, a marker of cycling cells^34^. Fewer cells treated with the inhibitor were positive for Ki67 than control cells (Fig. 1E), suggesting that, indeed, exposure to Trametinib mainly causes antiproliferative effects in GB cell lines. This notion is further supported by the increase of Trametinib-treated cells in the G_0/1_ phase of the cell cycle when compared to control cells (Fig. 1F).

**Figure 2:**
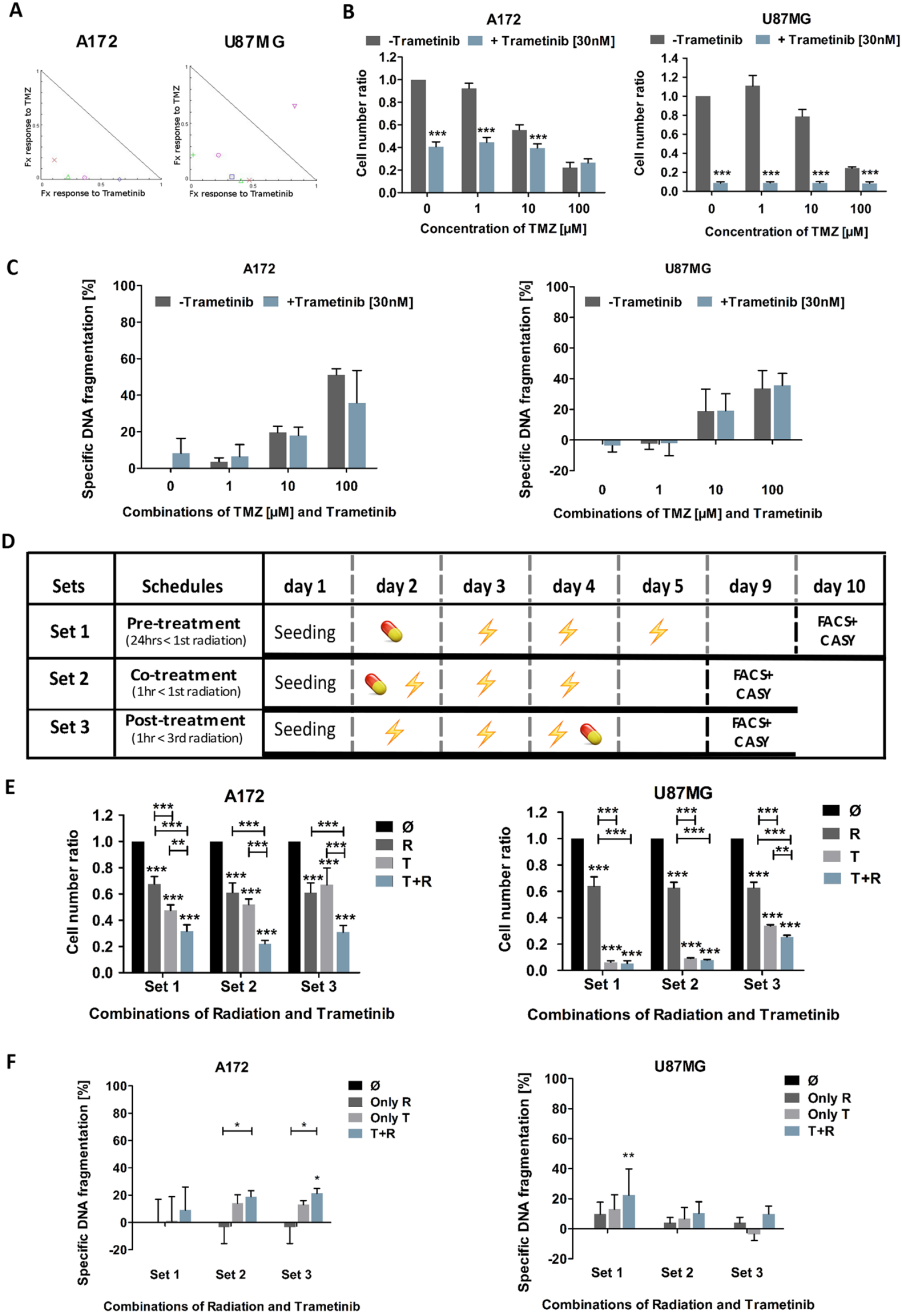
Evaluation of the MEK inhibitor Trametinib as part of a combination therapy approach with either chemo- or radiotherapy. (**A**) Effects on cell viability following treatment with Temozolomide (TMZ; at 1, 10 and 100 µM) and Trametinib (at 0.3, 3 and 30 nM) examined by MTT assay after 120 hours treatment. As three concentrations per drug were used, a total of nine distinct combinations could be examined, each combination is represented by a data point of unique colour/shape. Normalized isobolograms were calculated using the CompuSyn software tool. The connecting line represents additivity. Data points located below the diagonal line indicate a synergistic drug-drug interaction and data points above the diagonal line indicate an antagonistic drug-drug interaction. Data are obtained from three independent experiments performed in triplicate and summarized in Table 1. (**B**) Effect of combination of Trametinib and Temozolomide on the cell number of GB cell lines. The total viable cell number was measured using a cell counter after 120 hours of incubation of A172 (left) and U87MG (right) with 1, 10 and 100 μM Temozolomide in the presence or absence of 30 nM Trametinib as indicated. The control cells were treated with DMSO. The cell number ratio, normalised to controls, is defined as the ratio of cell number in treated population to cell number in the respective control. (**C**) Effect of Trametinib and Temozolomide combination on induction of apoptosis in GB cell lines. Flow cytometric analysis of Propidium-iodide stained nuclei after 120 hours of treatment with 1, 10 and 100 μM Temozolomide in the presence or absence of 30 nM Trametinib in the GB cell lines A172 (left) and U87MG (right). Control cells were treated with DMSO. DNA fragmentation was taken as readout of apoptosis and treatment induced DNA fragmentation (specific apoptosis) was calculated as described in the methods section. (**D**) Schematic representation of the drug-irradiation schedules. For combination treatment of Trametinib and Irradiation, Trametinib was administered prior to (<) or after (>) irradiation with 2 Gray in the respective sets as indicated. (**E**) Effect of Trametinib in combination with irradiation on the cell number of GB cell lines. A172 (left) and U87MG (right) cells were treated with Trametinib, irradiation, or both in differently scheduled combinations as shown in C. Controls were treated with DMSO. The cell number was detected by cell counter after 120 hours always following the last fraction of irradiation. Depicted is the calculated ratio of the respective treatment to the respective control, i.e. control was defined as 1 for all three treatment sets. (**F**) Effect of Trametinib and irradiation combination on induction of apoptosis in GB cell lines. Flow cytometric analysis of Propidium-iodide stained nuclei of A172 (left) and U87MG (right) cells after treatment with Trametinib, irradiation, or both in differently scheduled combinations as indicated in (**C**). Controls were treated with DMSO. In (**B**) mean and +SD of three independent experiments performed in sextuplicate are shown, in (**C,E,F**) mean and +SD of three independent experiments performed in triplicate are shown. Statistical significance was assessed by ANOVA (*p-value < 0.05; **p-value < 0.01; ***p-value < 0.001).

To test whether Trametinib also potentially functions as a sensitizer to an apoptosis-inducing stimulus, we next exposed the cells to the alkylating agent Temozolomide (TMZ), the standard chemotherapy used to treat GB patients. Using 30 nM or lower concentrations of Trametinib and combining this with physiological relevant concentrations of TMZ^35^, we could demonstrate that certain combinations of both substances can synergize in their effect on cellular metabolism (Fig. 2A, Table 1). However, these combinations did not markedly further reduce cell numbers (Fig. 2B). Similarly, combining both substances, which are, at physiological concentrations, not potent inducers of cell death, does not increase the overall apoptosis rate, as assessed by DNA fragmentation, compared to single substance treatment (Fig. 2C).

**Table 1:** Combination indices (CI) for A172 and U87MG glioblastoma cells treated with the indicated concentrations of Temozolomide (TMZ) and Trametinib (Tram).

TMZ [µM]	Tram [nM]	CI [A172]	CI [U87MG]
1.0	0.3	n/a	2.00473
1.0	3.0	418.991	65.3043
1.0	30.0	2.98444	**0.40387**
10.0	0.3	**0.25625**	1.47692
10.0	3.0	1.23495	4.08104
10.0	30.0	**0.65564**	**0.47712**
100.0	0.3	**0.29330**	**0.25038**
100.0	3.0	1.52078	**0.45116**
100.0	30.0	**0.38133**	**0.36958**

Note: synergistic interactions are bold.

Our recent work looking at radiation-based combination therapy in GB cells suggests that the temporal sequence of treatment can affect the outcome significantly^12^. As our data so far indicate that Trametinib treatment has a predominantly antiproliferative effect affecting the cell cycle distribution which, in turn, mediates sensitivity to radiotherapy^36^, we devised three different sequence combinations of inhibitor treatment followed by three doses of 2 Gray, mimicking the clinical situation during the radiotherapy phase of GB therapy^12^. These schedules are summarized in Fig. 2D. Similar to TMZ treatment, we observed an effect on cell numbers by both radiation and Trametinib treatment, but combining both approaches did not further enhance efficacy (Fig. 2E). Induction of apoptosis is also not enhanced by combining the inhibitor and radiation (Fig. 2F).

Taken together our data suggest that exposure of GB cell lines to the MEK inhibitor Trametinib leads to a reduction of cells over time when compared to a vehicle-treated control population. As total cell numbers remain relatively stable, cells only undergo little apoptosis and a clear reduction of Ki67-postive cells, as well as an increase of cells in the G_0/1_ phase of the cell cycle was observed after treatment, we concluded that Trametinib mainly works by being antiproliferative, most likely causing a G_0_ arrest. When combining Trametinib with the standard treatment for GB, the alkylating agent TMZ or radiation, we observed little sensitisation, suggesting MEK inhibition is not a good sensitizer to be used in combination therapy. However, it should be pointed out that the inhibitory effect on cell numbers was partially stronger after Trametinib treatment than that observed after standard therapy. Furthermore, Trametinib did not antagonize the apoptosis-inducing effect of TMZ or radiation treatment.

### MEK inhibition potentially leads to a more aggressive phenotype in Glioblastoma (GB) cell lines

**Figure 3:**
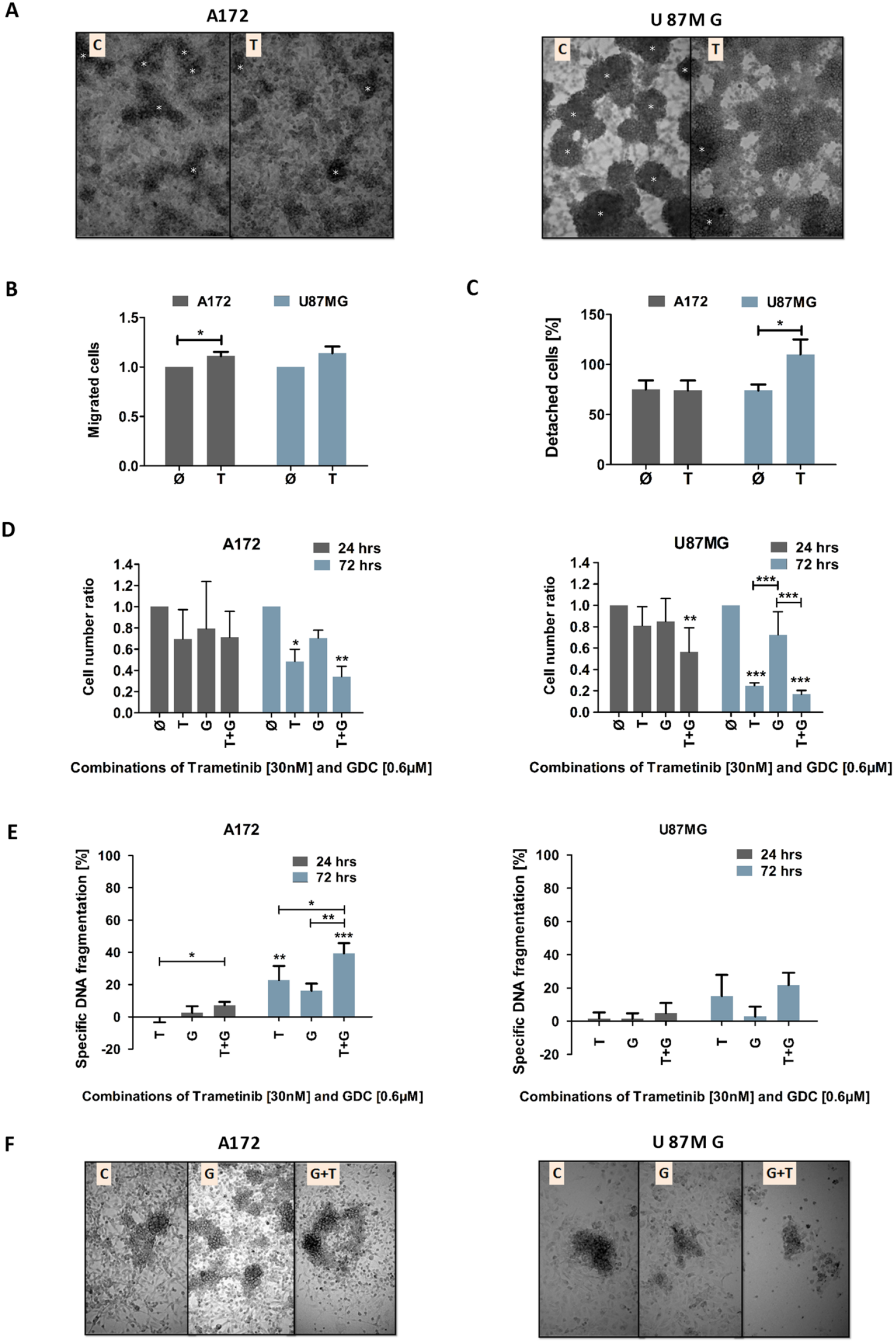
The influence of MEK inhibition on cellular motility and evaluating the combination of Trametinib and the PI3K-inhibitor GDC-0941. (**A**) Effect of Trametinib on sprouting. Representative pictures of A172 (left) and U87MG (right) cells showing the switch from cell-cell to cell-substrate interaction after transfer as described in Material and Methods and being exposed to Trametinib or solvent; C: control cells, T: Trametinib-treated cells. White asterisks indicate large cell clusters that resemble in size those initially transferred to new plates containing serum-enriched serum. (**B**) Effect of Trametinib on cell invasion. The migration of A172 and U87MG cells through a collagen-covered membrane was investigated in the absence of a chemoattractant gradient. Cells were pretreated with solvent or Trametinib for 24 hours and allowed to invade through the membrane for eight hours. (**C**) Effect of Trametinib on adhesion. The adhesion of A172 and U87MG cells in the presence of trypsin was assessed. Shown is the percentage of cells that detached after 5 minutes compared to 10 (for A172) or 20 (for U87MG) minutes, when cells were deemed to have fully detached. (**D**) Effect of combination of Trametinib and Pictilisib (GDC-0941) on the cell number of GB cell lines. The total viable cell number was measured using a cell counter after 72 hours of incubation of A172 (left) and U87MG (right) with 30 nM Trametinib, 0.6 μM Pictilisib (GDC-0941), or a combination of these two inhibitors. The control cells were treated with DMSO. The cell number ratio, normalised to controls, is defined as the ratio of cell number in treated population to cell number in the respective control. (**E**) Effect of Trametinib and Pictilisib (GDC-0941) combination on induction of apoptosis in GB cell lines. Flow cytometric analysis of Propidium-iodide stained nuclei after 72 hours of treatment with 30 nM Trametinib, 0.6 μM Pictilisib (GDC-0941), or a combination of these two inhibitors. in the GB cell lines A172 (left) and U87MG (right). Control cells were treated with DMSO. DNA fragmentation was taken as readout of apoptosis and treatment induced DNA fragmentation (specific apoptosis) was calculated as described in the methods section. (**F**) Effect of Pictilisib (GDC-0941) or Pictilisib (GDC-0941) and Trametinib on sprouting. Representative pictures of A172 (left) and U87MG (right) cells showing the switch from cell-cell to cell-substrate interaction after transfer as described in Material and Methods and being exposed to Trametinib or solvent; C: control cells, T: Trametinib-treated cells. In (**A,F**) a representative result of three independent experiments performed in triplicate is shown, in (**B**) mean and +SD of three independent experiments performed in duplicate are shown, while in (**C**–**E**) mean and +SD of three independent experiments performed in triplicate are depicted. Statistical significance was assessed by ANOVA, except for (**B,C**) were a two-tailed t-test was employed (*p-value < 0.05; **p-value < 0.01; ***p-value < 0.001).

While performing the experiments described above, we noticed how cells in the presence of Trametinib appeared to exhibit an altered morphology. To further analyse this perceived difference, we investigated the cells’ ability to switch between dominant forms of interaction. In particular, we employed the sprouting assay to observe the change between predominantly cell-cell-based interactions to cell-substrate-based interaction. This simulates the different microenvironments encountered by motile, invading cells and cells found within the tumour bulk^10,37^. Interestingly, cells which had been exposed to Trametinib for a prolonged time more readily left the cell clusters to spread out between individual high-density colonies (Fig. 3A). This reflects a more mobile and, therefore, potentially a more invasive phenotype. To further elucidate this potential phenotype, we employed a transmigration assay, where cells invade through a collagen-covered membrane. Here, too, prolonged exposure to Trametinib can lead to an increase in invasion (Fig. 3B), while additional experiments also demonstrated that the adhesive strength of Trametinib-treated cells can also be altered (Fig. 3C).

As we had previously observed that inhibition of the PI3K signalling cascade, which is closely intertwined with MEK signalling, also can have an antiproliferative effect, but in contrast to Trametinib inhibits cell motility, we next sought to combine PI3K and MEK inhibition. While the PI3K cascade is the prototypically activated survival pathway in GB and many therapeutic approaches attempt to utilize this for combination therapies^6–8^, the antiproliferative effect of Pictilisib, a PI3K inhibitor, does not exceed that of Trametinib, and combining both substances does not significantly improve the effect of the individual substances (Fig. 3D). This appears to be different when considering apoptosis, where combination treatment slightly, but significantly increases cell death induction compared to the individual substances (Fig. 3E). Importantly, the presence of Pictilisib appears to prevent the sprouting of Trametinib-treated cell clusters (Fig. 3F).

Taken together our data suggest that the antiproliferative effect of Trametinib on GB cell lines comes at a price: the cells develop a more aggressive phenotype in terms of motility and, therefore, potentially become more invasive. This cellular behaviour is often referred to as “Go or Grow” dichotomy^38^. Inhibition of PI3K signalling, which is also antiproliferative (although to a lesser extent than MEK inhibition), but also reduces motility^11,13^, blocks the increased sprouting of Trametinib-treated GB cell clusters and slightly increases the overall cell death but has no discernible effect on overall cell numbers.

### Glioblastoma (GB) cell lines accurately predict the effects of Trametinib on GB stem cell-line cells (SCs) and differentiated cells (DGBCs)

**Figure 4:**
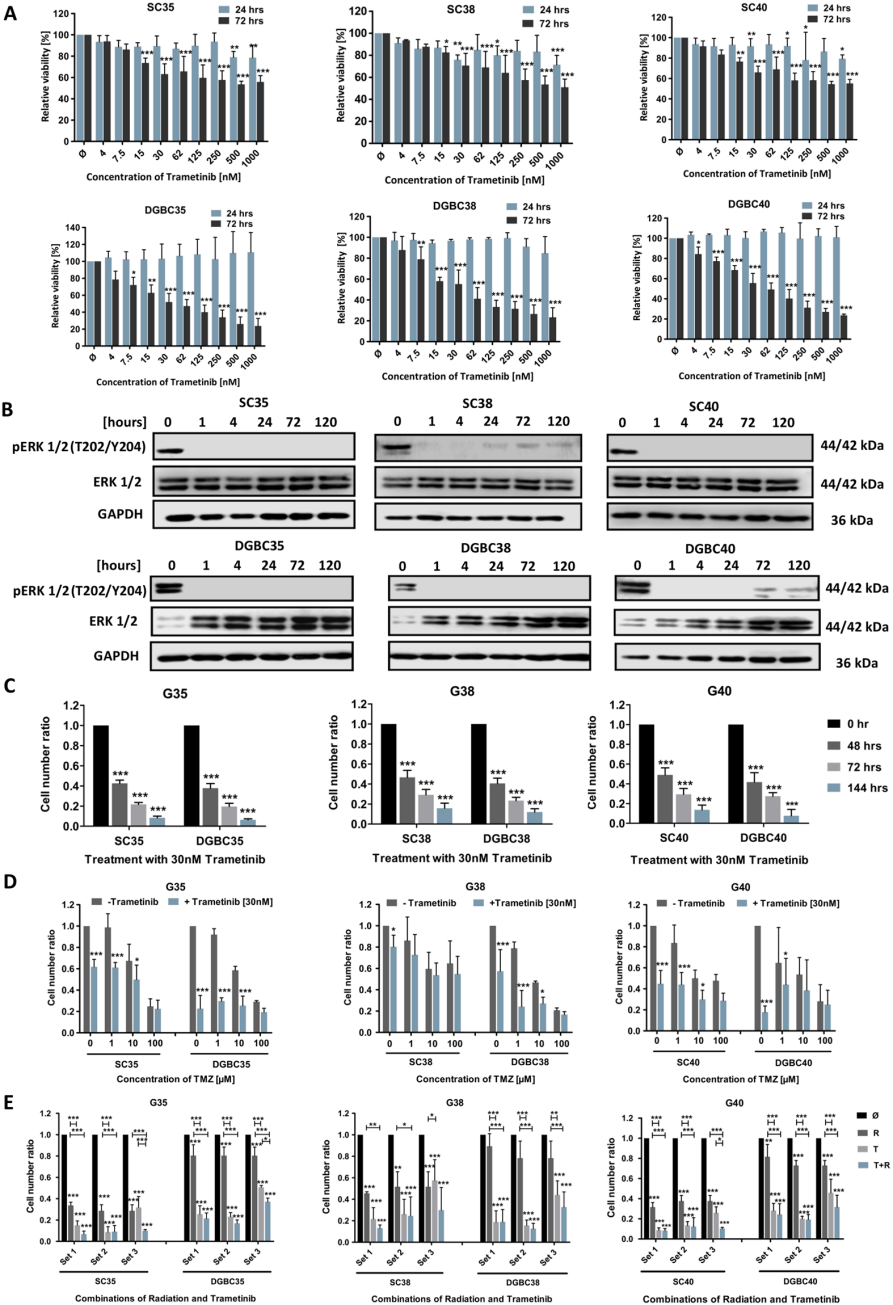
Evaluating MEK inhibition in GB stem cell-like cells and differentiated cells. (**A**) Effect of Trametinib on cell viability of GB primary material. Shown are the MTT assay results for three stem cell-like cell (SC) populations (upper row) and the corresponding short-term differentiated GB cell (DGBC) population (lower row). The cells were treated with indicated concentrations of Trametinib and the metabolic activity was measured after 24 and 72 hours. Data was normalized to the control. (**B**) Effect of Trametinib on signalling proteins in GB primary cultures. Activity of the MEK signalling cascade was assessed by Western blot analysis using phosphorylation of ERK as surrogate readout for activity of the MEK/ERK pathway. The SCs (upper row) and DGBCs (lower row) were treated with 30 nM of the MEK inhibitor Trametinib for the indicated times. GAPDH served as loading control. (**C**) Effect of Trametinib on cell number in GB primary cultures. The number of viable SC and corresponding DGBCs was measured using a cell counter at 24, 72 and 120 hours after treatment with 30 nM Trametinib. The control cells were treated with DMSO. The cell number ratio was defined as the ratio of cell number in the treated population to cell number in the respective control. The cell numbers at 0 hour were considered to be equal for the control and treated and hence taken as 1. (**D**) Effect of combination of Trametinib and Temozolomide on the cell number of GB primary cultures. The total viable cell number was measured using a cell counter after 120 hours of incubation of SCs and the corresponding DGBCs with 1, 10 and 100 μM Temozolomide in the presence or absence of 30 nM Trametinib as indicated. The control cells were treated with DMSO. The cell number ratio, normalised to controls, is defined as the ratio of the cell number in treated population to the cell number in the respective control. (**E**) Effect of Trametinib in combination with irradiation on the cell number of GB primary cultures. SCs and the corresponding DGBCs were treated with Trametinib, irradiation, or both in differently scheduled combinations as shown in Fig. 2C. Controls were treated with DMSO. The cell number was detected by cell counter after 120 hours always following the last fraction of irradiation. Depicted is the calculated ratio of the respective treatment to the respective control, i.e. control was defined as 1 for all three treatment sets. In (**A**) mean and +SD of three independent experiments performed in sextuplicate are shown, in (**B**) a representative result of two independent experiments is depicted, in (**C**) mean and SD of three independent experiments performed in sextuplicate are shown, while in (**D,E**) mean and +SD of three independent experiments performed in triplicate are shown. Statistical significance was assessed by ANOVA (*p-value < 0.05; **p-value < 0.01; ***p-value 4 0.001).

It has been suggested that GB cell lines only poorly mimic the gene and protein expression of GB cells within a tumour^39,40^, while in an orthotropic mouse model GB cell lines grow encapsulated and non-invasive, being unable to recapitulate what one could considered a key feature of GB^41^. Isolated stem cell-like cells (SCs) from patient’s material and short term expanding these SCs to adherent differentiated cells (DGBCs) is a superior surrogate of the *in vivo* situation than established cell lines^39,42^. Therefore, we selected three pairs of previously characterized^13,41^ SCs and DGBCs and exposed these cells to Trametinib. The effects on metabolic activity of Trametinib are less pronounced in the slowly dividng^41^ SCs than in the fast dividing^41^ DGBCs (Fig. 4A). The SC/DGBC ratio for the population doubling times of 35 cells is 2.1, of 38 is 1.7, and of 40 is 1.9^13^; this suggests that MEK inhibition might strongly affect proliferation in GB cells. As the sensitivity of the established cell lines (Fig. 1A) lies between that of SCs and DGBCs, we continued with the same concentration of Trametinib, 30 nM. Next, we verified that ERK phosphorylation is also inhibited at the chosen concentration for at least 120 hours (Fig. 4B). Of note, here we also found differences between SCs and DGBCs, namely that in SCs both proteins, p42 and p44 are not equally phosphorylated and that only in DGBCs a compensatory upregulation of total protein occurs upon inhibition of phosphorylation (Fig. 1B). These data suggest that the MEK/ERK axis has different roles in SCs and DGBCs, again reflecting our previous findings regarding the PI3K pathway in GB cells^11^. Interestingly, the relative effect on cell numbers is consistent, i.e. similar in SCs and DGBCs, but also comparable across the three parings (Fig. 4C). However, similarly to the data obtained using the established GB cell lines, Trametinib did not further synergize with standard treatment modalities, such as TMZ (Fig. 4D) and radiation (Fig. 4E), to further reduce cell numbers.

**Figure 5:**
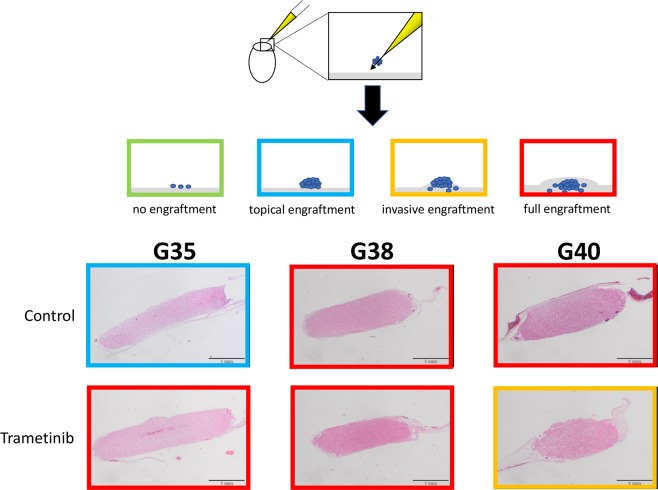
Effect of MEK inhibition on the invasion of differentiated cells into an organic membrane. The Chorioallantoic Membrane (CAM) assay is an attractive alternative to traditional *in vivo* assays regarding the assessment of angiogenesis and tumour invasion. The CAM is an extra-embryonic membrane that also contains extracellular matrix components. Shown in the scheme: Upon opening a fertilized chick egg, the CAM is exposed and tumour cells can be seeded upon it. These cells either do not engraft, topically engraft, engraft and begin to invade the underlying membrane, or – in the case of highly aggressive cells – fully invade the membrane. These different states are depicted by different coloured frame. Here, the CAM assay was utilized to study the invasive behaviour of differentiated G35, G38 and G40 cells on the CAM in the presence or absence of daily treatment with 100 nM Trametinib for four consecutive days. Pictures were taken after hematoxylin and eosin staining of slices made from tumour embedded paraffin blocks. The controls were treated with DMSO. The colour of the frame corresponds to the colour used in the above scheme depicting various states of engraftment. One representative picture out of each treatment group of six tumours is depicted here. Scale bar: 1 mm.

This data set suggests that established GB cell lines are a good surrogate to predict the effects of the MEK inhibitor Trametinib on GB SCs, as well as DGBCs. To finally look into the potentially motility-inducing effect that MEK inhibition might have on GB cells, we turned to the Chorioallantoic Membrane (CAM) assay, which is a good surrogate to ascertain the invasive behaviour of cells into a biological membrane. Here we could observe that tumours exposed to Trametinib can behave more invasive than GB cells treated with vehicle (Fig. 5). We therefore conclude that while MEK inhibition in GB cells potently reduces proliferation in a prolonged fashion, it does not further sensitize cells for standard therapeutic approaches and potentially can lead to an increased invasive behaviour. The latter aspect can be counteracted by concurrently blocking PI3K signalling.

## Discussion

Glioblastoma (GB), the most common primary brain tumour in adults, is characterized by an extremely aggressive phenotype, in essence, resembling a systemic brain disease^43^. Primary GB, while only rarely metastasising outside brain and central nervous system, has not been shown to exist in a pre-invasive form, hence tumour de-bulking, while alleviating symptoms, should not be considered a curative option^1,4^. Although radiation and chemotherapy in form of the alkylating agent Temozolomide (TMZ) further prolong average patient survival to about 15 months, full remission is exceedingly rare^3,44^. Novel therapeutic approaches, although urgently needed, are frequently hampered by the presence of the blood-brain barrier, which often blocks a dispersal of pharmacological substances throughout the brain.

When discussing contributory factors to the aggressive phenotype of GB, the focus has frequently settled on the PI3K/Akt/mTOR signalling axis, as it is activated in almost 90% of these tumours^6,7^. However, the contribution of PI3K-mediated signals to therapy resistance and motility are limited to distinct subpopulations within the tumour^11,13^ and modulation of this signalling cascade has not led to significant clinical successes^8^. It has been suggested that signal pathways can be rerouted^45^ and, particularly in GB, a high flexibility of compensatory resistance mechanisms might exist, an effect which has been dubbed the ‘Nile Distributary Problem’^9,46^. Therefore, we looked at a signalling network related and interlinked with the PI3K cascade, which affects similar cellular aspects of aggressiveness as PI3K signalling does, i.e. proliferation, motility and survival, yet independent enough so that inhibition of PI3K would not necessarily negatively affect signalling through this route. The MEK/ERK pathway is one potential signalling network that should be further investigated, particularly as a clinically approved inhibitor, Trametinib, could readily be included in any GB treatment schedule.

Alterations in the v-raf murine sarcoma viral oncogene homolog B (BRAF) protein, the upstream activator of the MEK-ERK signalling cascade, are frequently found in both, paediatric and adult brain tumours, but extremely rare in GB, present in only 1–2% of these tumours^47^. However, the absence of activating mutations in this pathway in GB should not be taken as an indicator for a reduced importance. It is worth bearing in mind that MEK/ERK signalling is activated by receptor tyrosine kinases, such as the Epidermal Growth Factor Receptor (EGFR)^48^. Enhanced EGFR activity by gene amplification or mutation is found in more than 57% of all GBs^49^, reducing the potential need for additional activating mutations further downstream of the signalling cascade.

Blocking MEK signalling in GB – established cell lines, as well as stem cell-like and differentiated cells – is clearly antiproliferative as it prevents an increase in cell number and reduces the percentage of cells positive for the cell cycle marker, Ki67, while also altering the cell cycle distribution. However, the absence of MEK activity, did not cause cell death *per se* and neither did it greatly sensitize cells for apoptosis induced by the current standard therapeutic interventions for GB, TMZ and radiation. This is in line with recent data that showed a similar lack of sensitisation for TMZ by MEK inhibitors of a previous generation, U0126 and PD98059^50^. Of note, these older inhibitors need to be administered in high concentrations^51^ and are often associated with increased toxicity due to the elevated amounts of solvent present during the analyses and thus cannot be considered for clinical application.

Importantly, as the presence of Trametinib does not antagonise the effects of treatment and exerts an antiproliferative effect, the net result of combination treatment can still be an overall reduction of tumour burden. However, we also observed an altered morphology of treated cells and when investigating the underlying cellular changes associated with this altered morphology, we found that GB cells in the presence of Trametinib more readily leave a cell cluster to spread out, i.e. the change in dominant form of interaction, moving from cell-cell to cell-substrate adhesion, occurs more readily when MEK is inhibited. This is not necessarily surprising, as it has long been described that the highly motile phenotype of GB cells comes at a price. Hypoxia, often induced by lack of vascularisation or rapid proliferation, leads to activation of genes responsible for motility, i.e. induces a migration to ‘greener pastures’. Concurrently, proliferation is reduced in those invading cells. This mechanism is referred to as ‘the cost of migration’ or ‘go or grow’^4,38^ and is probably best studied in the context of epithelial-to-mesenchymal transition (EMT). As already mentioned, the invasive nature of GB is ubiquitous^4^ and as such no traditional EMT occurs, indeed all GB subtypes, and not only the mesenchymal, exhibit a similar expression pattern to that found in epithelial cells that have undergone EMT^52^. Treatment has been shown to further increase the EMT signature in GB^53^ and can lead to more aggressive tumour behaviour^54^. Interestingly, exposure to Bevacizumab, a monoclonal antibody that blocks vascular endothelial growth factor (VEGF)-mediated signalling, has been associated with increased incidences of distant or diffuse disease^54^. ERK signalling can also be activated by VEGF^55^, and there is evidence for complex interaction between VEGF, EGF, ERK and PI3K^56^. The adverse events of Bevacizumab treatment might be blocked by adding further signalling modulators, such as blocking the Src family^57^, or inhibiting Integrins^58^.

While combination therapies can also modulate the adverse effect of MEK inhibition, for example by adding an inhibitor of PI3K, surely such a combination, which is exquisitely sensitive to drug concentrations achieved at different brain regions at different times, is only acceptable if the substance exhibiting adverse effects is of high therapeutic potency. Unfortunately, we have no evidence that this is true for MEK inhibition in the majority of GB cells. MEK inhibition does not generally synergize with cell death inducers, as shown by us and others^50^ and its antiproliferative effect is not necessarily stronger than that induced by PI3K inhibition; the latter has the additional advantage of not being pro-invasive. However, a recent report indicates a promising response to Trametinib by a Neurofibromatosis type 1 (NF1) - associated GB patient after tumour recurrence^59^. This suggests that Trametinib could be an effective drug, at least in a subtype of GB, like NF1-associated GB, where RAS-MAPK signalling is found to be amplified. Furthermore, the inability of Trametinib to potently sensitize GB cells for cell death, might be due to its inability to break the intrinsically high resistance to apoptosis exhibited by GB cells. ERK is described to affect cell death via modulation of the Bcl-2 family^60^, which only plays a relative minor role in GB^61–64^ compared to, for example, in leukaemia, where blocking Bcl-2 can be sufficient to induce apoptosis^65^. However, ERK is also involved in immune-resistance/immune-escape of cancer cells^60^, a potential interesting therapeutic avenue not addressed by this work.

In summary, while our data suggest that modulation of MEK signalling is not a strategy superior to inhibition of PI3K signalling and has additional pitfalls which would need to be managed. There might be a subgroup of GB where this treatment option is appropriate and further work is needed before discharging Trametinib as a potential contributor to combination therapy for GB.

## Methods

### Cell culture

The Glioblastoma cell lines A172 and U87MG were purchased from the American Type Culture Collection (ATCC, Manassas VA, USA), while the primary Glioblastoma stem cell-like cell populations (SCs) were isolated from patient tumour material as previously described^41,66^. SCs were cultured as non-adherent spheres in T75 non-tissue culture flasks (Sarstedt, Nümbrecht, Germany) utilising DMEM/F-12 (HAM) medium (Gibco, ThermoFisher Scientfic, Waltham, MA, USA), supplemented with EGF (Biomol GmbH, Hamburg, Germany), FGF (Miltenyi Biotec GmbH, Bergisch Gladbach, Germany), B27 (Gibco) and Penicillin/Streptomycin (Pen/Strep). Differentiated GB cells (DGBCs) were induced by culturing SCs in DMEM (Gibco), supplemented with 10% foetal calf serum (Gibco) and Penicillin/Streptomycin (Gibco) using T75 tissue culture flasks. Newly differentiated cells were passaged at least four times under differentiation conditions before used for experiments and passaged to a maximum of 10 times. The cell lines were cultured similarly to DGBCs.

### Reagents and irradiation source

Trametinib (GSK1120212) – Selleckchem, Housten, Texas, USA

Temozolomide (TMZ) – Sigma-Aldrich, Steinheim, Germany

Pictilisib (GDC-0941) – Selleckchem

The irradiation device used (HWM D400) was manufactured by Wälischmiller Engineering GmbH (Markdorf, Germany).

### Metabolic activity assay

The readout for viability was metabolic activity, which was assessed by an MTT assay as previously described^13^.

### Western blot analysis

Western blot analysis was performed as previously described^13^, and following antibodies were used:

Mouse anti-p-ERK 1/2 -p44/42 MAPK(Thr202/Tyr204) – Cell Signaling, Frankfurt, Germany

Rabbit anti-ERK 1/2 – Sigma-Aldrich

Rabbit anti-p-Akt (S473) – Cell Signaling

Mouse anti-Akt – BD Bioscience, Heidelberg, Germany

Mouse anti-GAPDH – Hytest Ltd., Turku, Finland

For chemiluminescent visualisation goat anti-mouse IgG or goat anti-rabbit IgG conjugated to horseradish peroxidase at 1:5000 (Santa Cruz Biotechnology, Heidelberg, Germany) were used.

### Determination of apoptosis

DNA fragmentation was used as a surrogate readout for apoptosis and assessed by flow cytometric (FACScan, Becton Dickinson, Heidelberg, Germany) analysis of propidium iodide-stained nuclei as previously described^13^. Specific apoptosis was defined as: 100 × (experimental DNA fragmentation [%] - spontaneous DNA fragmentation [%])/(100% - spontaneous fragmentation [%]).

### Determination of the number of viable cells

The number of viable cells at a given point in time was determined by counting cells using CASY1 DT (OMNI Life Science, Bremen, Germany), as previously described^13^.

### Fluorescence microscopy

Cells were treated as previously described^13^ and stained with a mouse anti-Ki-67 antibody (Dako Deutschland GmbH, Hamburg, Germany), which in turn was visualized using an FITC-labelled secondary antibody (Santa Cruz Biotechnology). Pictures were taken with an AX70 ‘Provis’ microscope (Olympus, Hamburg, Germany).

### Sprouting assay

The cell lines were seeded in 24-well plates at a density of 0.5 × 10^5^ cells/cm^2^ in 0.1% FCS medium to allow semi-adherent sphere formation. After 48 hours at 37 °C the cells were treated with Trametinib or solvent and transferred to a 6-well plate. After incubation for 24 hours at 37 °C the cell sprouting was detected using a CK40 microscope (Olympus). The same set-up was used for combination treatment. Instead of Trametinib alone, the cells were treated with GDC-0941 alone or a combination of GDC-0941 and Trametinib.

### Invasion assay

Cells were treated with Trametinib for 24 hours prior to seeding 5,000 cells on a collagen-covered transmembrane migration (Transwell®) insert. Transwell® membranes (Corning Incorporated, Corning, NY, USA) with a pore size of 8 μm were used, while collagen type I was obtained from BD Biosciences, Bedford, MA, USA. Eight hours after seeding cells were removed from the top layer of the insert and the remaining cells were fixed with 3.7% Paraformaldehyde and stained with 4′6-diamidino-2-phenylindole (DAPI) prior to mounting. Cells were then counted under the microscope.

### Adhesion assay

The cell lines were seeded in 24-well plates at a density of 0.5 × 10^5^ cells/cm^2^ and allowed to adhere overnight. Following 24 hours of exposure to solvent or Trametinib, medium was removed and a Trypsin/EDTA solution (Biochrom AG, Berlin, Germany) was added. Cells that had detached at indicated time points were counted using a CASY1 DT cell counter (Innovatis, Reutlingen, Germany).

### Chorioallantoic membrane assay

The invasion of cells into a biologic structure and the therapeutic response within a three-dimensional microenvironment was assayed via the chorioallantoic membrane (CAM) assay, as previously described^67^. Sections were stained with hematoxylin and eosin.

### Statistical analysis

Statistical analysis was carried out by a two-way ANOVA, followed by a Bonferroni post-hoc test for multiple group comparisons. Combination indices and isobolograms were calculated using CompuSyn software (ComboSyn, Inc., Paramus, NJ, USA).

## Data Availability

Non-commercial materials and data are available upon request.
